# Texture, color, and sensory changes occurring in chocolate bars with filling during storage

**DOI:** 10.1002/fsn3.2434

**Published:** 2021-07-13

**Authors:** Luděk Hřivna, Lenka Machálková, Iva Burešová, Šárka Nedomová, Tomáš Gregor

**Affiliations:** ^1^ Department of Food Technology Faculty of Agronomy Mendel University in Brno Brno Czech Republic; ^2^ Department of Food Technology Faculty of Technology Tomas Bata University in Zlín Zlín Czech Republic

**Keywords:** chocolate bar, fat bloom, retemperation, storage

## Abstract

The impact of storage temperature (6, 12, 20, 30℃) and period (2, 6, 10, 18, 26 weeks) on the texture, color, and sensory characteristics of dark and milk‐filled chocolate bars was studied. Temperatures of 6 and 12℃ were the most suitable for bar storage; these samples were evaluated not to be significantly deteriorated by the storage period. The condition of samples stored at 20℃ started to deteriorate after 10 weeks in storage; the decline was observed mainly in meltdown rate and off‐flavors, resulting in low overall acceptability. This effect was more evident in dark chocolate bars. Keeping the bars at 24℃ for 24h immediately after production (retemperation) improved the bar resistance to fat bloom, even if the decrease in the sensory quality was observed at the beginning of the storage period.

## Graphical abstract

This paper is focused on the impact of the production technology and storage conditions on chocolate bar quality. Temperatures of 6 and 12℃ were the most suitable for bar storage. Keeping the bars at 24℃ for 24h immediately after production (retemperation) improved the resistance of bars to fat bloom formation.

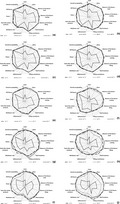

## 1 INTRODUCTION

Chocolate is a semi‐solid suspension of fine solid particles of sugar, cocoa, and milk powder (depending on the type), making about 70% total, in a continuous fat phase, consisting mostly of cocoa butter (Afoakwa, 2014). Chocolate shelf‐life is limited by the changes in the polymorphic state of cocoa butter or by rancidity development in solid chocolate. The resistance of chocolate to fat bloom is closely related to the crystallization of cocoa butter (Fernandes et al., 2013). At least six crystal forms (I‐IV) of cocoa butter can be distinguished, however, form V is most desirable. This form can be achieved by performing proper tempering procedures (Fernandes et al., 2013; Quast et al., 2013). In a traditional tempering process, the chocolate is first heated to about 50℃ to melt all present cocoa butter crystals and then cooled to about 27℃ to start the crystallization process. At this stage, the chocolate contains butter in form V; unstable polymorphs are also present. Unstable crystals melt, leaving only form V crystals in the form of seeds in the tempered chocolate after re‐heating to about 29–32℃ (Lonchampt & Hartel, 2004; Quast et al., 2013).

Oil from fats or oils within other components (fillings) may migrate and recrystallize on the surface as bloom. The stability of the product is also affected by physical properties of the nonlipid ingredients (size, shape, and surface chemistry of sugar, dairy solids, and cocoa solids). Lipid and ethanol migration from the filling into the chocolate shell is a key factor affecting the shelf‐life of composite products (Subramaniam, 2000). Lipids migration is typically observed when the chocolate shell or coating is in direct contact with lipid‐rich components like nuts, biscuits, or cream fillings. While lipid migration may result in bloom, the shelf‐life is often limited by textural changes, especially softening of the chocolate shell and hardening of the filling (Ziegler, 2009). Ethanol migration may negatively impact chocolate shell stability since ethanol dissolves the substances from the chocolate shell. This may result in the shell weakening and the evaporation of the ethanol (Talbot, 2009).

The shelf‐life of composite chocolate products may be limited, especially if they are subjected to unfavorable storage temperatures (Couzens & Wille, 1997). Keeping storage temperatures below the melting point of confectionery fat is not sufficient to prevent bloom formation because of the narrow melting range of confectionery fats and the significant impact of slight changes in temperature on solid content. Keeping chocolate products under 18℃ is, generally, recommended to minimize bloom formation (Widlak & Hartel, 2012). Storage at low temperature (T < 18℃) generally minimizes bloom formation. Storage in the 18–30℃ temperature range is below the melting point of Beta V crystals of cocoa butter and bloom occurs more quickly with an increase in temperature. When the temperature reaches 32℃, the cocoa butter is partially melted. Upon subsequent cooling, the cocoa butter crystallizes uncontrollably into unstable polymorphic forms, causing bloom to occur very quickly (Ali et al., 2001; Wooton et al., 1970).

Chocolate shelf‐life may be prolonged if chocolate is seeded with a large amount of stable crystals in form V. It can be expected that the amount of these crystals may be increased by keeping the chocolate below the melting point of the stable cocoa butter crystals (retemperation). These temperature conditions made the removal of latent heat and the creation of a crystal network of the V form possible. Hence the aim of the study was to evaluate the impact of keeping chocolate bars immediately after production for 24 hr at 24℃ (retemperation) on the changes occurring during a 6‐month storage period. The effect of different storage conditions (6℃, 12℃, 20℃, and 30℃) on texture, color, and sensory characteristics was also evaluated. Dark and milk chocolate bars with different fillings were involved in this study to compare the impact of selected factors on different products.

## 2 MATERIAL AND METHODS

### 2.1 Material

The research was performed on chocolate bars kindly provided by Zora Olomouc (Nestlé Česko s.r.o.). *ORION Kaštany ledové* (45 g) is a dark chocolate bar with cocoa‐hazelnut filling (shell 47%, filling 53% w/w). Ingredients: sugar, cocoa mass, vegetable fats (palm, palm kernel), skimmed milk powder, 4% cocoa powder with reduced fat, cocoa butter, hazelnut paste 1.6%, vegetable fats, milk fat, emulsifiers (sunflower lecithin, polyglyceryl polyricinoleate), whole milk powder, whey powder, citric acid, flavorings. Cocoa solids content of dark chocolate was at least 40%. *ORION Milena* (32 g) is a milk chocolate bar with rum‐flavored filling (shell 50% w/w, filling 50% w/w). Ingredients: sugar, vegetable fats (palm, palm kernel), cocoa butter, whole milk powder, cocoa mass, glucose syrup, vegetable fats, milk fat, lactose, whey powder, emulsifiers (sunflower lecithin, polyglyceryl polyricinoleate). Cocoa solids content in milk chocolate was at least 25%.

The chocolate bars were produced using a standard production process (Meyer, 2009). The production process was completed by wrapping bars individually in polypropylene foil and storing them at 18℃ for 24 hr. After this, the products were moved to the thermostats and stored at (6, 12, 20, and 30 ± 1) °C for 6 months. The selected temperatures represented cooling conditions (6℃ and 12℃), room temperature (20℃), summer conditions, and warmer climate (30℃). These samples were marked with the letter N. A half of produced samples was kept at 24℃ for 24 hr between wrapping and storing at 18℃. Keeping the samples at 24℃ was expected to enhance crystal growth (Campos & Marangoni, 2012). This step was described as retemperation (R). The retempered samples were, subsequently, also stored at 6, 12, 20, and 30℃ for 6 months.

Before testing, each sample was equilibrated over a 24‐hr period at room temperature (20 ± 1℃). The characteristics of the 8 samples stored at the each temperature were evaluated. The obtained results are presented as mean values.

### 2.2 Hardness of chocolate bars

Material testing machine TIRAtest 27,025 (TIRA GmbH, Germany) equipped with a 200 N load cell was used to test the hardness of the chocolate bars according to De Clercq et al., (2017). The cylindrical probe (diameter 5 mm) moving by a speed of 2 mm/s penetrated into the chocolate bar. The penetration distance was set to 5 mm. The maximum load was defined as the hardness. The testing was performed in an air‐conditioned laboratory at a temperature of 20 ± 1℃. Hardness evaluation was performed immediately after production and 2, 6, 10, 18, and 26 weeks after production.

### 2.3 Color determination

Color of the chocolate was determined using a Konica Minolta Spectrophotometer CM 3500D (Konica Minolta Business Solutions Czech, lnc. Czech Republic). The measurements were performed using an 8‐mm diaphragm.

The CIELAB color space was determined using CM‐S100w SpectraMagic NX software. Spectrophotometer measurements of color in the visible region represent a suitable complement to sensory analysis. The lightness L* represented the darkest black as L* = 0, and the brightest white as L* = 100. Color coordinates a* and b* represented neutral gray values as *a* = 0 and b* = 0. The red/green colors were represented along the a* axis with green as negative a* values and red as positive a* values. The yellow/blue colors were represented along the b* axis with blue as negative b* values and yellow as positive b* values. The sample color was measured in triplicates on the shell and in triplicates on the coating. The color of two samples stored at the same temperature was evaluated. The bar color was tested immediately after production, 2, 6, 10, 18, and 26 weeks after production.

### 2.4 Sensory evaluation

Optimized Descriptive Profile (Santos Navarro et al., 2013) was used to evaluate the sensory attributes of chocolate bars. The products stored at different temperatures were tested at the same time to make comparing the samples possible. The panelists (8) were both male and female at the age of 19–55 years, selected from postgraduate students and staff. The panelists were trained according to the ISO 8586–1 standard (1993). The sensory evaluation was performed under standard conditions (ISO 8,589). The graphic scaling consisted of an unstructured 10‐cm horizontal linear ruler, marked at both ends by a legend. The attribute of intensity/acceptability increased from left to right. The value of the attribute was obtained by measuring the distance between the left end of the scale and the mark. The attributes of appearance (color, gloss, absence of fat bloom in the shell, coating, and filling), texture (hardness, consistency of the filling, adhesiveness, and meltdown rate), taste, flavor, off‐flavors, and overall acceptability were evaluated. The sensory evaluation was performed immediately after production, 2, 6, 10, 18, and 26 weeks after production. The mean values are present in radar charts.

### 2.5 Statistical evaluation

Statistical evaluation was performed using STATISTICA 12.0 software (TIBCO Software Inc.) using a multifactor analysis of variance (ANOVA). The significance of the differences was tested using the Tukey test (α = 0.05).

## 3 RESULTS AND DISCUSSION

The study was performed to evaluate the changes in dark and milk chocolate bars initiated by storing temperature and period together with the impact of keeping chocolate bars at 24℃ for 24 hr before storing (retemperation).

### 3.1 Bar hardness

**FIGURE 1:**
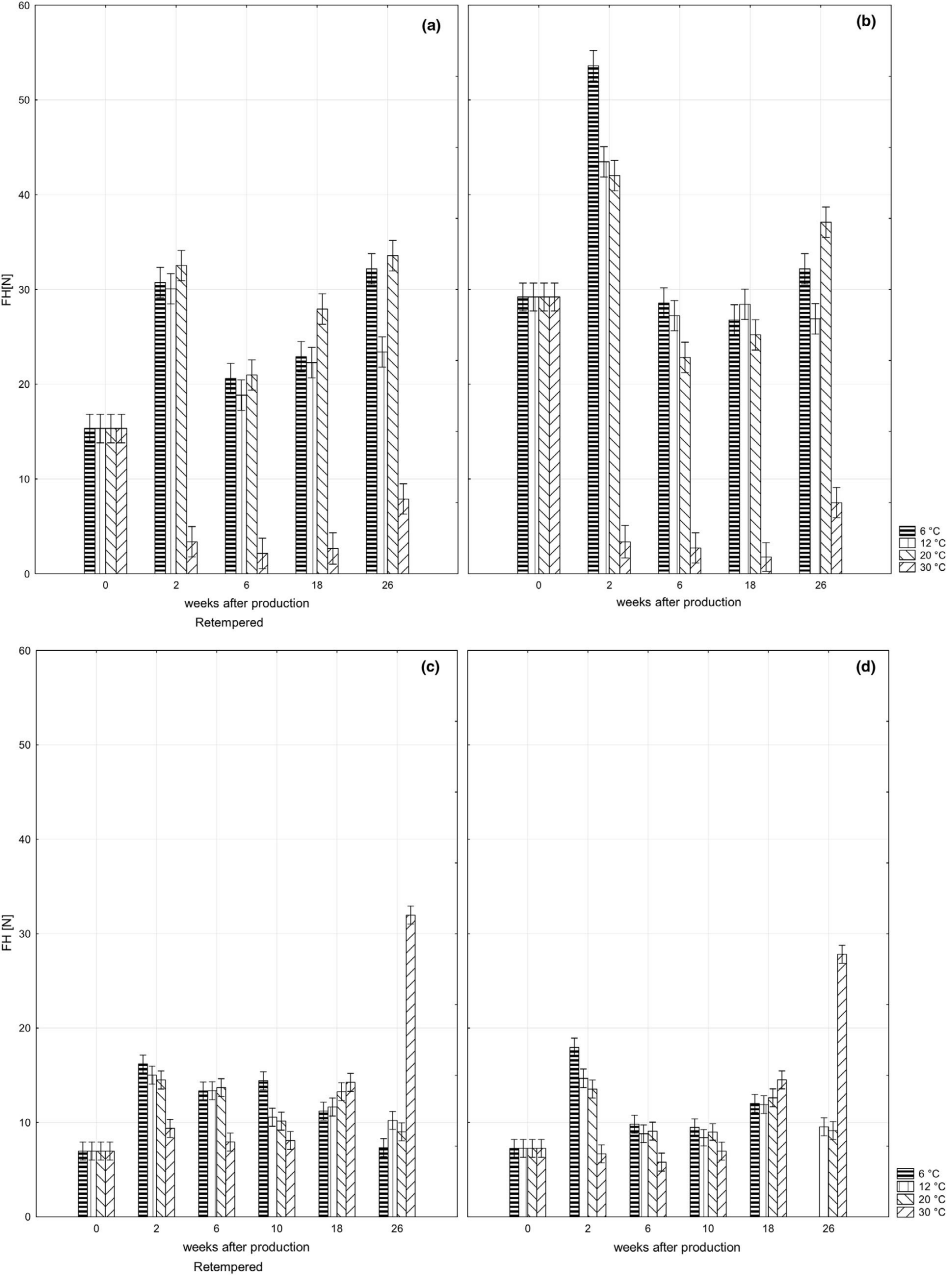
Changes in bar hardness during storage: retempered samples of dark chocolate bars *ORION Kaštany ledové* (a), dark chocolate bars *ORION Kaštany ledové* produced using a standard production process (b); retempered samples of milk chocolate bars *ORION Milena* (c); milk chocolate bars *ORION Milena* produced using standard production process (d)

Chocolate hardness is one of the important mechanical characteristics as it affects sensory acceptability of the product. Keeping a dark chocolate bar *ORION Kaštany ledové* at the temperature of 24℃ for 24 hr immediately after production (retemperation) resulted in significantly (*p* <.05) lower values of hardness than in a bar produced using standard production technology (Figure 1a,b).

Low hardness indicates that the retemperation created better conditions for the formation of larger particles/crystals of cocoa butter than standard technology (Afoakwa et al., 2008a). The impact of retemperation was obvious after 2 weeks in the samples stored at the temperature of 20℃. The lower storage temperatures (6 and 12℃) prolonged this effect to up to 10–18 weeks. These results indicated the time and temperature dependence of particle/crystal of cocoa butter formation occurring during storing (Afoakwa et al., 2007; Ali et al., 2001). The disappearance of the differences between retempered and standard samples during storage may be a result of processes toward the unique stable size of the particle/crystal of cocoa butter, regardless of production technology.

The hardness of milk chocolate bars *ORION Milena* were significantly (*p* <.05) lower than in dark bars due to the presence of milk fat‐inhibiting cocoa butter crystallization (Metin & Hartel, 2012). The retemperation did not impact the hardness of this bar immediately after production (Figure 1c,d). After 6 and 10 weeks of storing, the hardness of retempered milk chocolate bar was, however, higher than in the samples produced by standard technology. The different impact of retemperation on dark and milk chocolate bars at these phases of storage may be related to the presence of milk fat causing an eutectic effect, influencing the tempering process and the changes in fat crystals directly related to bar hardness (Afoakwa, 2014; Ali et al., 2001). The differences in hardness of retempered and standard milk chocolate bar disappeared after 18 and 26 weeks of storing. A similar trend was described for dark chocolate bars.

The impact of storage at 30℃ on the hardness of dark and milk chocolate bars varied. The hardness of dark chocolate bars was significantly (*p* <.05) decreased two weeks after production and remained at almost the same level during all storage periods. The decrease of bar hardness may be related to it approaching the melting point of fat (Ostrowska‐Ligęza et al., 2019). The hardness of milk chocolate bars stored at the same temperature, however, significantly increased after 18 and 26 weeks of storage. The values of hardness reflect the texture of the chocolate shell together with the characteristics of bar filling since the probe penetrated 6 mm into the bar and the thickness of the chocolate shell varied between 1–2 mm. The differences in the results obtained in dark chocolate bar and milk chocolate bar may be, therefore, related to the changes in particle size occurring in the fillings due to their different contents.

### 3.2 Bar color

**FIGURE 2:**
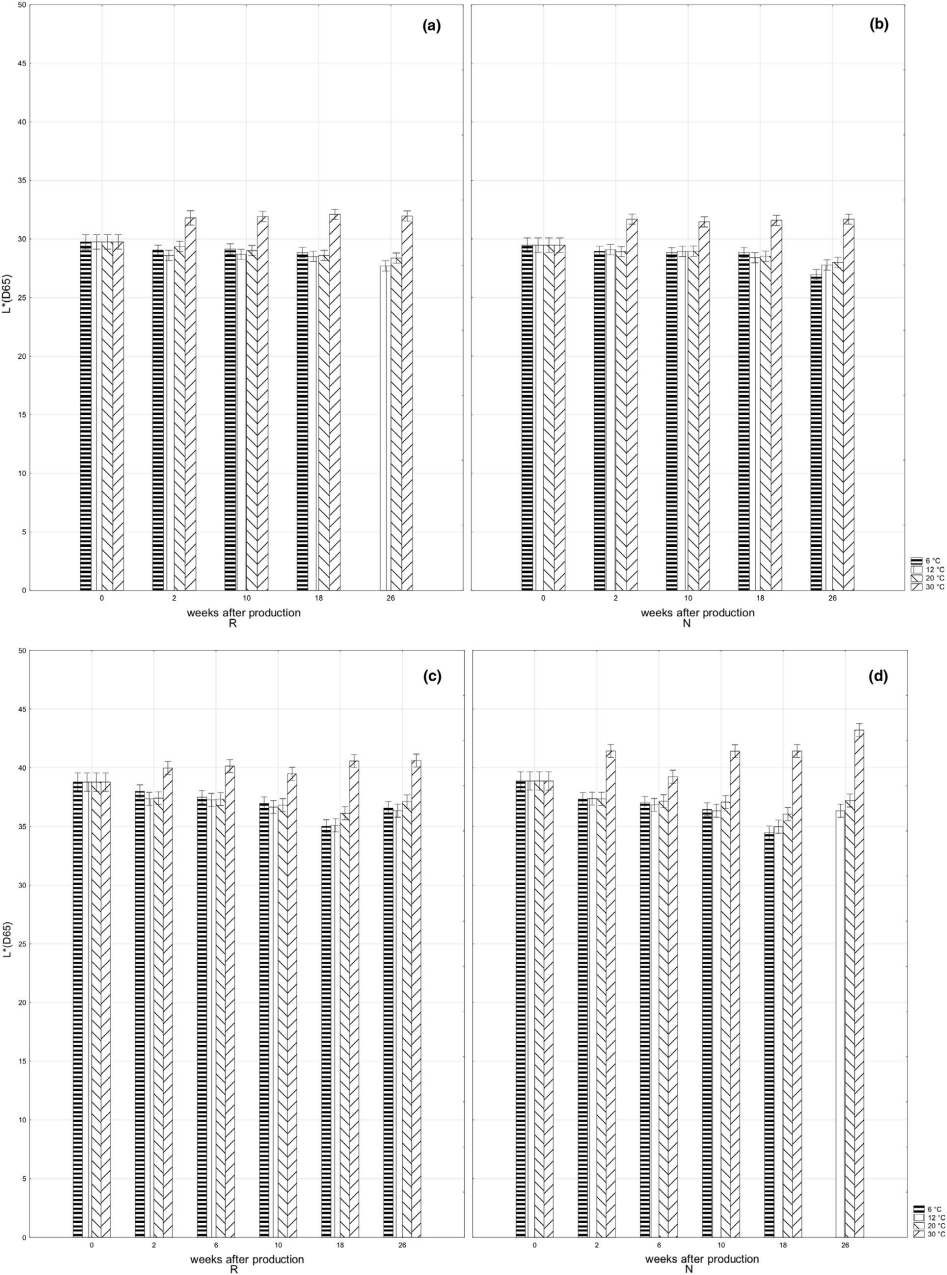
Changes in bar lightness L* during storage: retempered samples of dark chocolate bars *ORION Kaštany ledové* (a), dark chocolate bars *ORION Kaštany ledové* produced using a standard production process (b); retempered samples of milk chocolate bars *ORION Milena* (c); milk chocolate bars *ORION Milena* produced using a standard production process (d)

**FIGURE 3:**
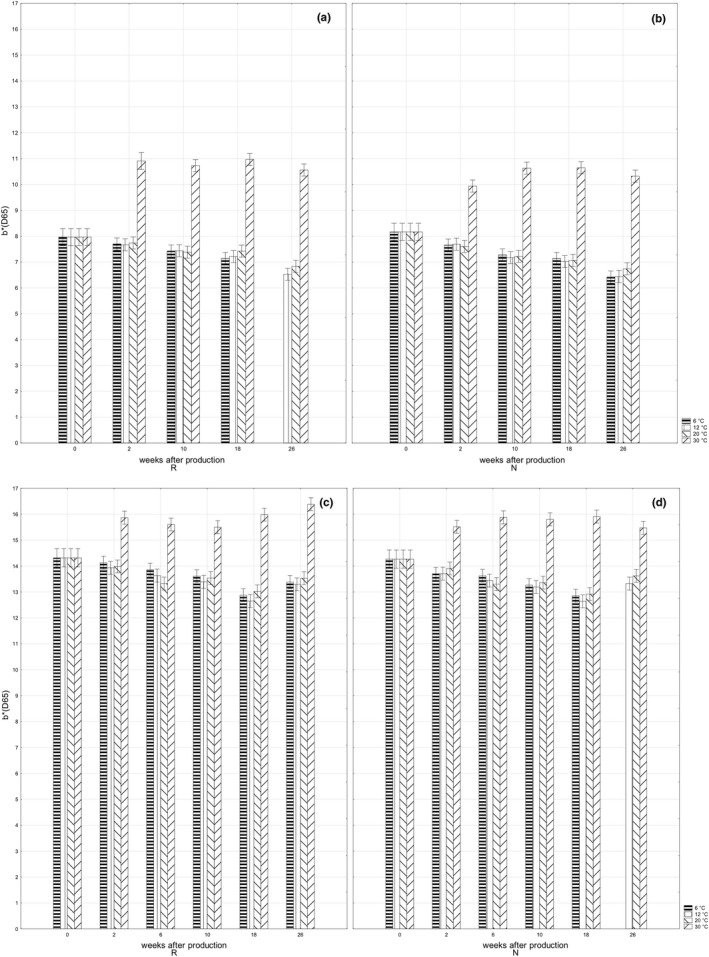
Changes in bar color b* during storage: retempered samples of dark chocolate bars *ORION Kaštany ledové* (a), dark chocolate bars *ORION Kaštany ledové* produced using a standard production process (b); retempered samples of milk chocolate bars *ORION Milena* (c); milk chocolate bars *ORION Milena* produced using a standard production process (d)

The values of lightness L* recorded in dark chocolate bar *ORION Kaštany ledové* stored at 6, 12, and 20℃ were nearly constant or exhibited a slight decrease toward the end of the storage period (Figure 2a,b); the decrease of b* values was most evident (Figure 3a,b). The obtained values were, moreover, similar in retempered and standard samples. It is obvious the color of dark chocolate bars stored at 6, 12, and 20℃ was stable during the storage period and the color was not affected by the retemperation. The increase in L* and b* values recorded in dark chocolate bars stored at 30℃ was inversely related to bar hardness. The softer bars appeared lighter, indicating bloom formation on the bar surface (Afoakwa et al., 2008b; Briones & Aguilera, 2005; Zhao & James, 2019).

*ORION Milena* is produced from milk chocolate, therefore, the values of lightness L* and b* higher than in dark chocolate bar were the expected results (Figure 2a,b,c,d; Figure 3a,b,c,d). The retemperation of bars at 24℃ for 24 hr after production did not influence the color of the products before storing (Figure 2c,d; Figure 3c,d). The color of the bars stored at the temperatures of 6, 12, and 20℃ got darker during the 18‐week long storage period, which is evident from the decreasing values of L* (Figure 2c,d). The previously described relation between the changes of the lightening and particle size (Afoakwa et al., 2007, 2008b) indicates a rising particle/crystal size in milk chocolate bar. This explanation is supported by the softening of milk chocolate bar observed during a 10‐week storage period, since chocolate softening can be related to a rising size of cocoa butter crystals (Afoakwa et al., 2008b). The decrease in hardness was slightly less extensive in retempered samples, indicating the retemperation stabilized the particle/crystal size in milk chocolate. The color shifting to white, observed as increasing values of L* after 2 weeks of storage at the temperature of 30℃, may be explained by melting cocoa butter and fat leaking from the inner parts onto the surface of the milk chocolate bar (Briones & Aguilera, 2005).

### 3.3 Sensory attributes of the bars

**FIGURE 4:**
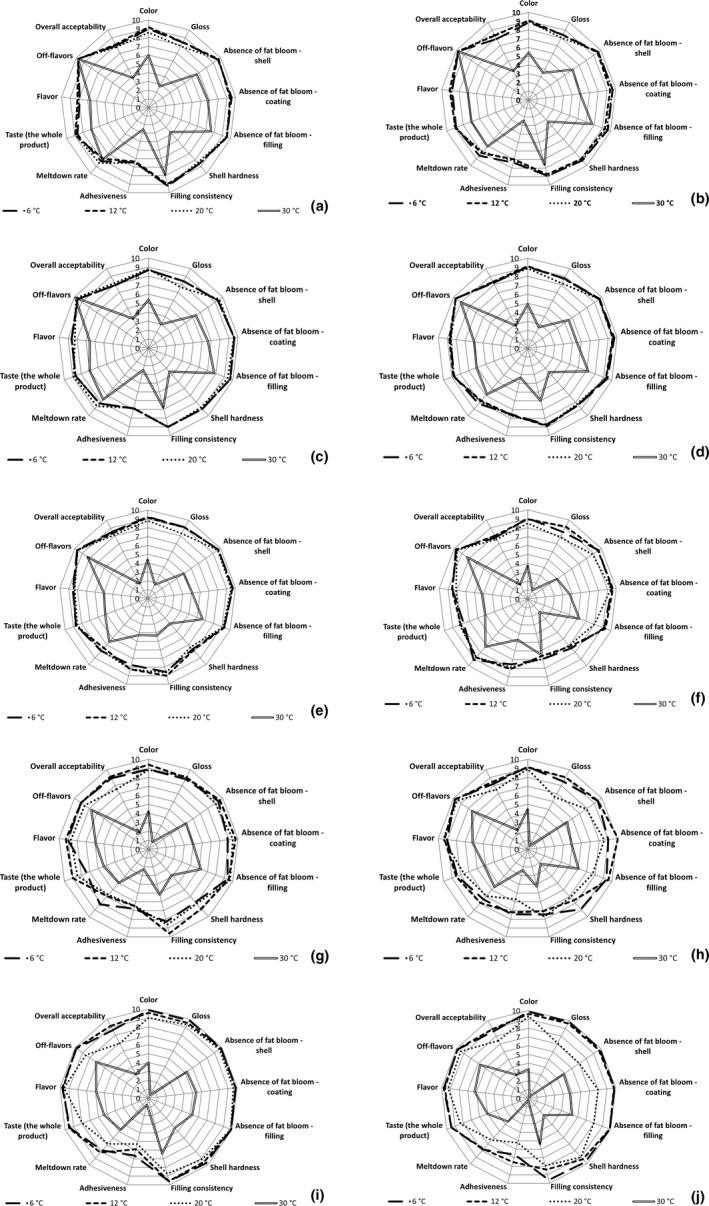
Changes in sensory evaluation scores of dark chocolate bar *ORION Kaštany ledové*: retempered samples 2 weeks after production (a), bars produced using a standard production process 2 weeks after production (b), retempered samples 6 weeks after production (c), bars produced using a standard production process 6 weeks after production (d), retempered samples 10 weeks after production (e), bars produced using a standard production process 10 weeks after production (f), retempered samples 18 weeks after production (g), bars produced using a standard production process 18 weeks after production (h), retempered samples 26 weeks after production (i), bars produced using a standard production process 26 weeks after production (j)

**FIGURE 5:**
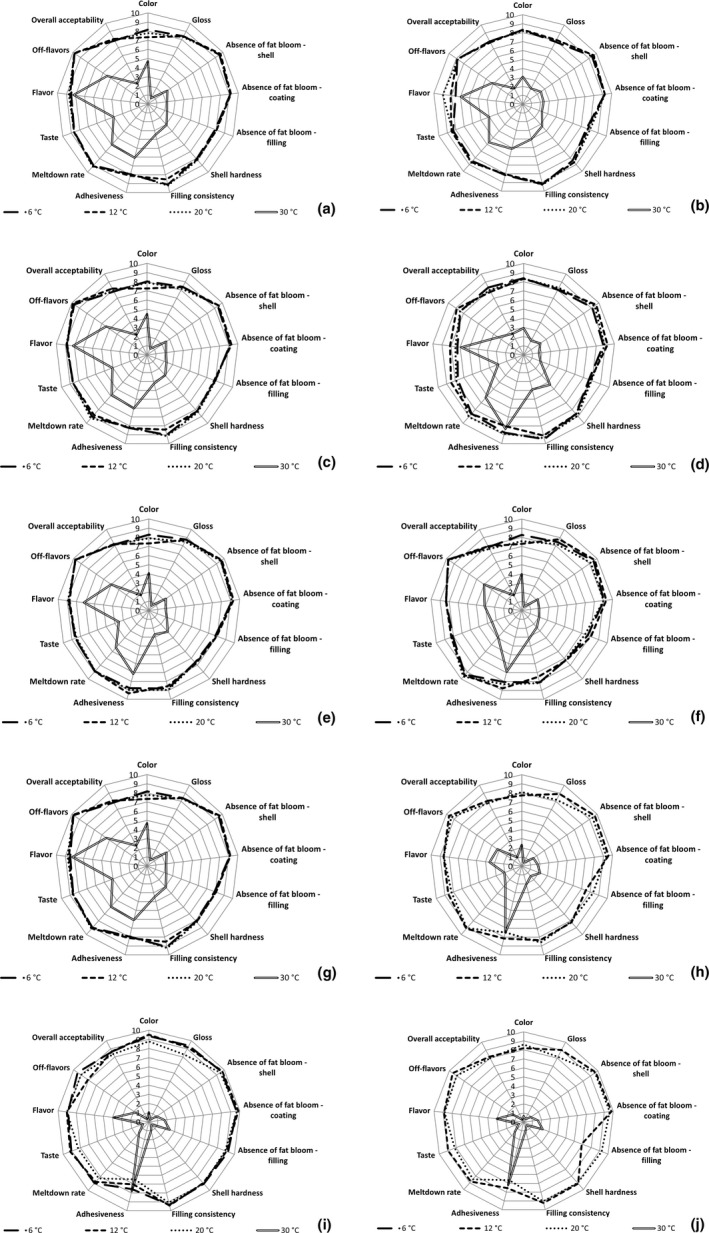
Changes in sensory evaluation scores of milk chocolate bars *ORION Milena*: retempered samples 2 weeks after production (a), bars produced using a standard production process 2 weeks after production (b), retempered samples 6 weeks after production (c), bars produced using a standard production process 6 weeks after production (d), retempered samples 10 weeks after production (e), bars produced using a standard production process 10 weeks after production (f), retempered samples 18 weeks after production (g), bars produced using a standard production process 18 weeks after production (h), retempered samples 26 weeks after production (i), bars produced using a standard production process 26 weeks after production (j)

The initial sensory evaluation did not show any impact of keeping the bars at 24℃ for 24 hr (retemperation) on the attributes of dark and milk chocolate bars (data not included). The decrease of hardness observed by TIRAtest in the retempered dark chocolate bar *ORION Kaštany ledové* immediately after production was not perceived by the panelists. The panelists, however, recognized slight differences during the evaluation performed after 2 weeks (Figure 4a,b; Figure 5a,b) in the samples stored at 6, 12, and 20℃. The hardness of retempered bars was scored slightly lower (8) than the harder standard product (9). The subsequent significant decrease of bar hardness recorded by TIRAtest after 10 weeks of storage was negatively perceived by the panelists, which is evident from the lower score of shell hardness (7–8) (Figure 4e,f). The subsequent increase in bar hardness recorded by TIRAtest was positively accepted by the panelists (Figure 4g‐j). The panelists found the hardness of dark chocolate bars stored for 26 weeks to be the most acceptable. It is evident that the evaluation of bar hardness was positively impacted by the storage period. A similar improving impact of the storage period on the evaluation of shell hardness was recorded in milk chocolate bars stored at 6, 12, and 20℃ (Figure 5a‐j). The optimal hardness of dark bars was 23–37 N and 7–10 N in milk bars. The effect of retemperation on bar hardness was found mainly at the beginning of the storage period, lowering the bar evaluation scores. The impact of retemperation on sensory evaluation, however, disappeared with rising storage period (Figure 4a‐j; Figure 5a‐j), which is in agreement with the results obtained by TIRAtest.

The appearance of the bar surface determines the acceptance by the consumers. The decreasing of lightness L* and b* recorded by the spectrophotometer in the samples stored at 6, 12, and 20℃ was positively accepted by the panelists, which is evident from rising color and gloss scores (Figure 4a‐j; Figure 5a‐j). The effect was more evident in milk chocolate bars, which is in agreement with the more extensive changes of L* and b* recorded in milk chocolate bars. The impact of retemperation on bar color was not observed.

In samples stored at 6, 12, and 20℃, filling consistency and taste were negatively impacted by the 10‐week storage period. The effect was weaker in the retempered samples (Figure 4a‐j; Figure 5a‐j). The other parameters of the samples stored at 12℃ and 6℃ for 10 weeks did not exhibit significant differences from the values obtained after being stored for 2 weeks. The negative impact of storage temperature on the resistance of dark chocolate bars to fat bloom was detected in samples stored at 20℃ for 26 weeks (Figure 4j). This impact was, however, reduced in retempered dark chocolate bars (Figure 4i). The changes in surface appearance may be related to the increase of b* values recorded by the spectrophotometer that can be explained by the presence of yellowish cocoa butter and fat on the surface of the bar. Storage at the temperature of 20℃ for 26 weeks negatively impacted the evaluation of meltdown rate, and off‐flavors, resulting in the decrease of overall acceptability. This effect was more evident in dark chocolate bars (Figure 4a‐j; Figure 5a‐j).

The deteriorating impact of storage temperature was the most obvious in samples stored at 30℃. All tested attributes of the samples stored at 30℃ were significantly decreased by storage temperature. The sample evaluation scores decreased rapidly with longer storage periods (Figure 4a‐j; Figure 5a‐j). Due to the deteriorating impact of storage temperature, the panelists did not recognize the differences in retempered and non‐retempered bars. The panelists found the color, gloss, meltdown rate, and adhesiveness unacceptable. The taste and flavor was described as disgusting. The evaluation of milk chocolate bar *ORION Milena* was further decreased by saccharose crystals occurring in the filling. The presence of these crystals may be a possible explanation for the rapid increase of hardness detected in milk chocolate bar by TIRAtest after 18‐ and 20‐week storage periods.

## 4 CONCLUSIONS

The changes occurring in the studied bars were impacted by the type of chocolate (dark/milk), filling, storage temperatures, and storage period. The effect of retemperation was also observed. The significance of the changes initiated by retemperation, storage temperature, and period varied among studied samples. The storage temperatures of 6 and 12℃ were found to be the most suitable for storing dark and milk chocolate bars since the evaluation of these samples has not significantly deteriorated during the storage period. The negative impact of storage temperature on resistance to fat bloom was detected in samples stored at 20℃ for 26 weeks. This impact was, however, reduced in retempered dark chocolate bars. Storage at the temperature of 20℃ for 26 week negatively impacted the evaluation of meltdown rate and off‐flavors, resulting in the decrease of overall acceptability as well. The deteriorating impact of storage temperature was most obvious in samples stored at 30℃. The taste and flavor of these samples was described as disgusting. Moreover, saccharose crystals occurred in the filling. The temperature of 30℃ is not suitable for storing chocolate bars; the retemperation did not mitigate the negative impact of this temperature.

## CONFLICTS OF INTEREST

None.

## AUTHOR CONTRIBUTION

**Ludek Hřivna:** Conceptualization (lead); Investigation (lead); Methodology (lead); Project administration (lead); Supervision (lead); Writing‐review & editing (equal). **Lenka Machalkova:** Data curation (equal); Investigation (equal); Methodology (equal); Writing‐original draft (equal). **Iva Buresova:** Investigation (equal); Software (equal); Validation (equal); Writing‐original draft (equal); Writing‐review & editing (lead). **Šárka Nedomová:** Investigation (equal); Software (equal); Validation (equal); Writing‐review & editing (equal). **Tomas Gregor:** Conceptualization (equal); Funding acquisition (equal); Project administration (equal); Resources (equal).
